# Potassium Channel and NKCC Cotransporter Involvement in Ocular Refractive Control Mechanisms

**DOI:** 10.1371/journal.pone.0002839

**Published:** 2008-07-30

**Authors:** Sheila G. Crewther, Melanie J. Murphy, David P. Crewther

**Affiliations:** 1 School of Psychological Science, La Trobe University, Melbourne, Australia; 2 Brain Sciences Institute, Swinburne University of Technology, Melbourne, Australia; Vrije Universiteit Amsterdam, Netherlands

## Abstract

Myopia affects well over 30% of adult humans globally. However, the underlying physiological mechanism is little understood. This study tested the hypothesis that ocular growth and refractive compensation to optical defocus can be controlled by manipulation of potassium and chloride ion-driven transretinal fluid movements to the choroid. Chicks were raised with +/−10D or zero power optical defocus rendering the focal plane of the eye in front of, behind, or at the level of the retinal photoreceptors respectively. Intravitreal injections of barium chloride, a non-specific inhibitor of potassium channels in the retina and RPE or bumetanide, a selective inhibitor of the sodium-potassium-chloride cotransporter were made, targeting fluid control mechanisms. Comparison of refractive compensation to 5mM Ba^2+^ and 10^−5^ M bumetanide compared with control saline injected eyes shows significant change for both positive and negative lens defocus for Ba^2+^ but significant change only for negative lens defocus with bumetanide 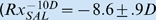; 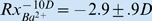; 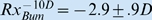; 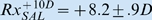; 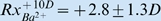; 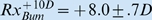). Vitreous chamber depths showed a main effect for drug conditions with less depth change in response to defocus shown for Ba^2+^ relative to Saline, while bumetanide injected eyes showed a trend to increased depth without a significant interaction with applied defocus. The results indicate that both K channels and the NKCC cotransporter play a role in refractive compensation with NKCC blockade showing far more specificity for negative, compared with positive, lens defocus. Probable sites of action relevant to refractive control include the apical retinal pigment epithelium membrane and the photoreceptor/ON bipolar synapse. The similarities between the biometric effects of NKCC inhibition and biometric reports of the blockade of the retinal ON response, suggest a possible common mechanism. The selective inhibition of refractive compensation to negative lens in chick by loop diuretics such as bumetanide suggests that these drugs may be effective in the therapeutic management of human myopia.

## Introduction

Myopic eyes in humans and animals are larger in axial dimensions and hence in volume than emmetropic eyes, while refractively hyperopic eyes are smaller overall than emmetropic eyes of the same age. The changes in axial dimensions and refraction are directly related to changes in vitreous chamber depth [1]. Refractive changes, approximately equal to the strength of blur experienced, can be induced in hatchling chicks with a few days of negative or positive optical defocus (reviewed [2]). Vitreal depth changes are also accompanied by inverse changes in the thickness of the choroidal vasculature such that optical defocus with negative lenses or form deprivation leads to increased vitreal depth and dramatic thinning of the choroid, while optical defocus with positive lenses is associated with shorter vitreous chamber dimensions and thickening of the choroid. How this balance between growth and refractive demand is achieved is not well understood. “Stop” and “Go” growth factors [3], choroidal control of the refractive plane [4] and scleral sculpting [5] have all been proposed as potential mechanisms of ocular growth. However, the problem of how the fluid necessary for ocular dimensional changes is accumulated or dispersed is only addressed by our theory of RPE/Müller cell controlled ionically driven trans-retinal fluid flow between vitreous chamber and the choroidal lymphatics [6].

Crewther [6] hypothesized that the increase in vitreous volume associated with form deprivation myopia was due to a reduction in the rate of the normal fluid outflow across the retinal pigment epithelium (RPE) from the retina to the choroid, with retention in the vitreous chamber. This model is based on four principles. The first, that the vitreous chamber and choroid act as fluid reservoirs in the eye, is supported by much previous ultrastructural research [7], [8]. The second, that the RPE controls movement of fluid between these reservoirs is also well supported by *in vitro* and *in vivo* physiological research (reviewed [9], [10]). The model further poses that reduction of temporal modulation of light under an occluder (a condition associated with myopia and abnormal ocular elongation) leads to reaccumulation of potassium (K) ions in the subretinal space (SRS) [11]–[14]. This predicted increase has since been reported [15]. The last principle–that fluid exchange across the RPE is causally driven by the refractive error signal, is yet to be proven. The proposed outer retinal/RPE site of determination of defocus and deprivation sensing is supported by various experiments that have interfered with inner retinal function without preventing refractive/growth compensation. These include optic nerve section [16], tetrodotoxin (TTX) elimination of spiking sodium channels (ganglion cells and some amacrines) [16] and NMDA inhibition of third order retinal neurons [17].

Elemental X-ray microanalysis of myopic form deprived (FD) chick retinas has shown an increase in atomic abundance of K, localized to the vicinity of the sub-retinal space (SRS), RPE and photoreceptor outer segments, and a generalized increase in the abundance of Na and Cl across the retina, RPE and choroid. Following the termination of FD, the high subretinal K abundance rapidly reduced to levels below normal until refractive recovery 5 days later [15], while Na and Cl abundances took several days to return to normal levels. Such increased abundance of potassium in the SRS leads to reduced SRS volume and reduces the movement of fluid across the RPE in *in vitro* preparations [18]. This implies that more of the fluid being synthesized in the retina as a result of glycolysis [19], [20] must remain in the extracellular space of inner retina or be transferred into the vitreous via the Müller cells. As the ultrastructural appearance of the FD retina is one of hyperosmolarity rather than edema, the latter alternative, of fluid movement into the vitreous, is the likely outcome. This conclusion is supported by the fact that less than 30min of normal visual experience following occluder removal leads to a substantial increase in choroidal thickness and the appearance of edema beginning to move across the retina from the vitreous towards the choroid [21].

Fluid movements across the RPE to the choroidal blood supply are controlled by the ionic channels, cotransporters and symporters of the apical and basal membranes of the RPE. *In vitro* eye cup studies demonstrate that numerous ionic species (K^+^, Na^+^, Ca^2+^, H^+^, Cl^−^, HCO_3_^−^, as well as lactate) affect subretinal space fluid dynamics (reviewed [22]). However, due to the fact that to date, only K, Na and Cl abundance have been experimentally associated with myopia [15], [21], and given that Crewther's [6] hypothesis related myopia to the activity of the inwardly rectifying K channels and the sodium potassium 2-chloride symporter (NKCC1) on the apical membrane of the RPE, we have chose to focus this study on potassium and the way in which potassium channels and cation-chloride transporters, particularly the sodium potassium 2-chloride symporter (NKCC1) on the apical membrane of the RPE, are intimately involved in the growth response to defocus.

NKCC1 belongs to the cation-chloride cotransporter family, which mediates the coupled movement of Na, K and two Cl ions in strict ratio across the plasma membrane of cells. NKCC1 transport is electroneutral, with the driving force for ion influx being in part supplied by the inward Na^+^ gradient and maintained by Na/K-ATPase [23]. These channels, symporters and exchangers, driven by alterations in K concentration, modulate fluid transfer by controlling the absorption and secretion of chloride [22], and hence water transport, via the cotransporters themselves [24] and in concert with aquaporin channels [19]. Sulfamoybenzoic acid loop diuretics such as bumetanide can compete with Cl^−^ for the second chloride binding site and thus inhibit NKCC1 function [23]. In fact when the kidney isoform of the NKCC cotransporter (NKCC2) is inhibited by bumetanide, a large increase in urine flow, nearly isosmotic with plasma, is observed, regardless of the hydration state of the individual. Thus, under diuretic treatment, an individual loses the ability to excrete either a concentrated or a dilute urine. The NKCC2 isoform, found in the thick ascending loop of Henle of the kidney, appears to rely on the recycling of K ions through luminal K channels for its normal operation, as barium blockade of the potassium channels significantly reduces Na reabsorption [25].

Cation-chloride transporters are found not only on epithelial cells, but also across the retina. Vardi et al [26] used antibodies to mGluR6 to label the metabotropic glutamate receptor at the photoreceptor ON-bipolar synapse, and showed that antibodies to NKCC1 but not KCC2 (potassium chloride symporter receptor protein) co-localize with the glutamate receptor. Similarly, anti-calbindin, that selectively labels an OFF-bipolar subclass, co-localizes with KCC2 but not NKCC1 immunoreactivity at the flat synapses of the photoreceptor OFF-bipolar interface. Thus KCC2 is expressed wherever the chloride equilibrium potential E_Cl_<E_rest_ (resting potential), whereas NKCC1 is expressed wherever E_Cl_>E_rest_ (ON bipolar dendrites). KCC2 was also found to be expressed on the OFF bipolar dendrites, ganglion cell and bipolar axons whereas NKCC1 antibodies typically labelled horizontal cells as well as the ON bipolar dendrites [26]. Thus, bumetanide, which is a selective blocker of NKCC1 may also alter retinal function by blocking the cation-chloride uptake at the ON-bipolar dendrites (and horizontal cells). Bumetanide also eliminates the directional responses of directionally selective ganglion cells and starburst amacrine cells [27], [28].

Given the likelihood that myopia induced by lens defocus shares many of the same mechanisms and ultrastructural changes as form deprivation [21], [29], it is probable that inhibiting potassium movements in such retinae would interfere with defocus induced refractive and growth changes, especially as apical potassium and basal chloride appear to be strongly linked in the RPE [30]. The RPE contains many different potassium channels. Early Ussing chamber experiments demonstrated the presence of weak inwardly rectifying potassium channels (K_ir_), with more recent research delineating the likely presence of K_ir_7.1 and K_ir_4.1 subfamilies (reviewed [22]). Despite their inwardly rectifying nature, the apical channels are largely involved in recycling K^+^ ions back to the SRS, a process that occurs because in RPE cells the resting membrane potential is more positive than the K equilibrium potential (E_Keq_), resulting in outward K^+^ currents. While Ba^2+^ blocks these K_ir_ channels with different sensitivities, it is also a non-specific blocker of voltage-gated and Ca^2+^-activated K^+^ channels [31]–[33]. Thus, in the first experiment we tested this idea by investigating the interaction of the non-selective potassium channel blocker Ba^2+^ with the induction of refractive error by lens defocus.

Loop diuretics, by comparison, act to inhibit the cation-chloride NKCC cotransporter, thus inhibiting the coordinated trans-membrane movement of Na, K and Cl ions [23]. Thus, in a second experiment, we tested the idea that if negative lens defocus results in myopia and ocular elongation through a reduction in the outflow of fluid across the RPE, then intravitreal injection of the diuretic bumetanide would restore RPE fluid flow as evidenced by relative suppression of abnormal vitreal chamber growth and inhibition of myopia.

## Results

### Experiment 1: Potassium channels and refractive compensation

**Figure 1:**
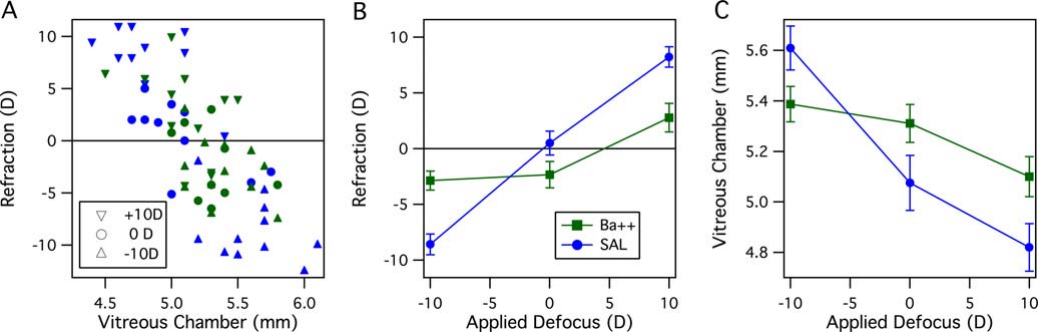
The effect on refractive and growth compensation of blocking retinal and RPE potassium channels with intravitreal Ba^2+^ ion at an effective concentration of 5mM. A. Scatter plot of Refraction in dioptres versus Vitreous Chamber depth (mm). across all eyes measured. The applied defocus is indicated by the triangles or circle symbols, with green symbols indicating Ba^2+^ and blue symbols indicating saline injected eyes. B. Refractions of eyes injected with Ba^2+^ compared with those injected with similar volume of Saline. Compensation in Ba^2+^ eyes is suppressed for both positive and negative lens defocus. The same colour code applies as for A. C. Vitreous chamber depths of Ba^2+^ and SAL eyes. An inverse relationship between vitreous chamber depth and refraction is evident. The same colour code applies as for A. The effect of defocus on VC depth is much less for Ba^2+^ than for SAL eyes, however the mean VC depth for Ba^2+^ eyes averaged over all lens groups is very similar to that for SAL eyes. This indicates that Ba^2+^ does not inhibit eye growth *per se*, but suppresses compensation to defocus-related eye growth. Data presented as means±SE.

Potassium channels were blocked via intravitreal injection of barium chloride to a vitreal concentration of approximately 5mM. Retinoscopy and ultrasonography demonstrated that while saline injected eyes showed about 85% refractive compensation to the applied defocus over the four days of rearing, barium suppressed refractive compensation to both positive and negative lenses, but did not significantly affect refractive state for the chicks reared with focused vision (0D group). A generally negative linear relationship between refraction and vitreous chamber depth is clearly evident in the data - across all lens groups and the two drug groups (Ba^2+^, SAL), the data fall along a main trend line (see Figure 1A). A between group Analysis of Variance (ANOVA) (for Lens Defocus (+10D, 0D, −10D) and Treatment (Ba^2+^, Saline)) demonstrated a significant main effect of Lens Defocus (*F(2,59)* = 61.1, *p*<.0001) but not of Treatment on ocular refraction (*F(1,59)* = 0.98, *p* = .32). A significant interaction between Lens Defocus and Treatment was also observed (F_2,59_ = 16.2, *p*<.0001), - see Fig 1B. Post-hoc tests showed the suppression of compensation to defocus by Ba^2+^, with significant refractive difference for both positive and negative lenses compared with saline injected eyes (Fisher's PLSD, *p*<.005) (see Table 1).

**Table 1:** Means Tables and Post-hoc comparisons Ba^2+^ vs SAL for Ocular Refraction and Vitreous Chamber Depth

	Rx Ba2+ (D)		Rx SAL (D)		Fisher's PLSD
Lens	Mean	SE	Mean	SE	*p*
−10D	−2.88	0.86	−8.59	0.93	0.0002
0D	−2.33	1.18	0.49	1.08	0.09 *ns*
+10D	2.77	1.28	8.23	0.91	<.0001

Lens	VC Ba2+ (mm)		VC SAL (mm)		Fisher's PLSD
	Mean	SE	Mean	SE	*p*
−10D	5.39	0.07	5.61	0.09	0.024
0D	5.31	0.08	5.08	0.11	0.11 *ns*
+10D	5.10	0.08	4.82	0.09	0.04

Similarly, ANOVA on vitreous chamber length showed the same pattern with significant main effect for Lens (*F(2,58)* = 20.8, *p*<.0001) but not for Treatment (*F(2,58)* = 1.94, *p* = .17), with a significant interaction between Lens Defocus and Treatment (*F(2,58)* = 5.5, *p* = .007) (see Fig 1C). Further, barium did not show a lens-dependant effect on anterior chamber depth (correlation rˆ2 = .048). Figure 1B indicates that an effective intravitreal concentration of 5mM barium chloride, which would be expected to non-selectively block potassium channels, inhibited refractive compensation for both positive and negative lens defocus but did not generally inhibit vitreal growth. A comparison of vitreal chamber lengths between Ba^2+^ and SAL eyes, taken across all lens groups, showed no significant difference (difference of means = 0.098 mm, Fisher's PLSD, *p* = .26) - see Table 1.

### Experiment 2: Effect of the loop diuretic bumetanide

**Figure 2:**
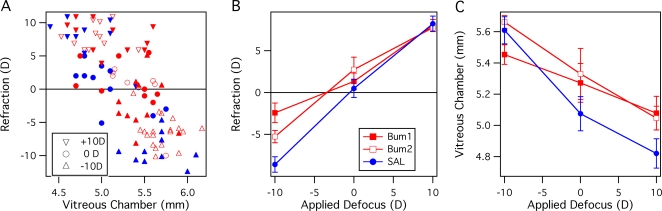
The effect on refractive compensation of the loop diuretic bumetanide at two concentrations in the vitreous chamber compared with control saline injections. Bum1 refers to an effective vitreal concentration of 10^−5^ M, Bum2 refers to an effective concentration of 5×10^−6^ M. A. Scatter plot of Refraction in dioptres versus Vitreous Chamber depth (mm) across all eyes measured. The applied defocus is indicated by the triangles or circle symbols, with red filled symbols indicating Bum1, red open symbols indicating Bum2 and blue symbols indicating saline injected eyes. B. Compensation to negative lens rearing markedly diminished for both bumetanide doses with the higher dose (Bum1) resulting in approximately 6D less refractive compensation than for SAL. The same colour code applies as for A. The refractive effect is specific to the induction of myopia, with 0D and +10D groups showing no significant effect of the intravitreal diuretic. C. Vitreous chamber depths of bumetanide and saline injected eyes. The same colour code applies as for A. While the same trend as in A is evident with a dose dependency with negative lens defocus, it can also be seen that mean VC depth is slightly larger for Bumetanide *cf* SAL eyes, especially for 0D and +10D groups. Data presented as means±SE.

In hatchling chicks, bumetanide suppressed refractive compensation to negative lenses in a dose specific fashion (here the two effective vitreal concentrations are referred to as Bum1 = 10^−5^M, Bum2 = 5×10^−6^M), but did not significantly affect refractive compensation to optical defocus of +10D or to zero power lenses (see Table 2). A scatter plot (Fig 2A) demonstrates the general negative linear relationship between refraction and vitreous chamber length. Closer inspection indicates that there may be small differences in this relation between drug groups. This is most easily observed for the Bum2 (open red symbols) compared with SAL (filled blue symbols), where linear regression shows parallel slopes of approximately −14D/mm, but with the Bum2 data offset by 0.18mm, i.e. with greater VC depth for the same refraction.

**Table 2:** Means Tables and Post-hoc comparisons Bum1, Bum2 vs SAL for Ocular Refraction and Vitreous Chamber Depth

	Rx Bum1 (D)		Rx Bum2 (D)		Rx SAL (D)		Bum1 v SAL	Bum2 v SAL
Lens	Mean	SE	Mean	SE	Mean	SE	PLSD *p*	PLSD *p*
−10D	−2.42	1.17	−5.27	0.74	−8.59	0.93	0.0001	0.01
0D	1.33	0.99	1.12	1.10	0.49	1.08	0.61 *ns*	0.68 *ns*
+10D	8.02	0.70	7.75	0.46	8.23	0.91	0.87 *ns*	0.69 *ns*

	VC Bum1 (mm)		VC Bum2 (mm)		VC SAL (mm)		Bum1 v SAL	Bum2 v SAL
Lens	Mean	SE	Mean	SE	Mean	SE	PLSD *p*	PLSD *p*
−10D	5.45	0.06	5.66	0.04	5.61	0.09	0.12 *ns*	0.53 *ns*
0D	5.27	0.12	5.37	0.08	5.08	0.11	0.17 *ns*	0.03
+10D	5.08	0.11	5.05	0.08	4.82	0.09	0.06 *ns*	0.08 *ns*

A between groups ANOVA (for Lens Defocus (+10D, 0D, −10D) and Treatment (Bum1, Bum2, Saline)) indicated significant main effects for Ocular Refraction of Treatment (*F(2, 98)* = 4.5, *p* = .0135), Lens Defocus (*F(2, 98)* = 177, *p*<.0001) and a significant Treatment*Lens Defocus interaction (*F(4, 98)* = 3.6, *p* <.01). At the higher concentration of bumetanide (Bum1: 10^−5^M), there was a significant difference in refractive compensation to negative lens defocus between the bumetanide and control Saline injected eyes (Bum1: −2.42±1.17 D, SAL: −8.59±0.93 D; Fisher's PLSD:, *p* = .0002–see Fig 2B). Rearing with −10D lenses, the higher dose of bumetanide resulted in a sparing of over 6 D of refractive compensation compared with the Saline group, while the lower dose was markedly less effective (−5.3±0.74 D). Post-hoc tests also indicate that the higher dose is significantly more effective than the lower dose (Fisher's PLSD: mean difference = 2.9 D, *p* = .0033). Indeed, further post-hoc testing within the separate lens rearing groups (see Table 2) indicates that bumetanide only significantly affects refractive state in the negative lens reared chickens.

Axial dimension of the vitreous chamber of the three negative lens wearing groups also show significant differences (see Fig 2C). ANOVA indicates a main effect of Treatment (2 doses of bumetanide, saline) (*F(2,96)* = 3.64, *p* = .03) and lens (*F(2,96)* = 30.1, *p*<.0001), while the Treatment*Lens Defocus interaction was not significant. Thus, eyes injected intravitreally with bumetanide, showed a slightly greater vitreal chamber depth after 4 days rearing than did eyes injected with the same volume of saline, measured over all Lens groups (see Table 2). Post-hoc comparisons within Lens defocus groups were not significant, except for 0D defocus with the lower dose of bumetanide. Anterior chamber depths were not affected in a sign-dependant fashion, with a low correlation found between AC depth and applied defocus (rˆ2 = .01).

The fellow eyes demonstrated similar refractive states across all drug and lens groupings of the experimental eyes. Anterior chamber depths of fellow eyes also did not show systematic differences with respect to the various experimental eye groups.

## Discussion

The experiments reported here demonstrate that both unselective blocking of potassium channels and selective inhibition of the sodium-potassium-chloride symporter can produce dramatic interference with refractive compensation to optically induced blur. As both subretinal [K] and the activity of the NKCC1 symporter of the apical membrane of the RPE (and the photoreceptor/ON bipolar synapse) are known to affect the shunting of fluid between the retina and the choroid [9], [11] these results are consistent with a mechanism of ocular growth and refractive error based on alteration in the rates of ionically controlled transretinal fluid flow, (particularly by Na, K and Cl, - the major ions of phototransduction and cell volume regulation), as originally hypothesized by Crewther [6]. A chief tenet of this theory of refractive and growth control is that alteration in outer retinal visual activity under conditions of defocus leads to changes in the ionic contents of the subretinal space (depending on the sign and degree of defocus). To date, the only published data relating myopia to ionic changes are restricted to K, Na and Cl abundances [15] . If, as postulated, [K^+^]_SRS_ is central to refractive control [6], [15], the effects of potassium on the RPE apical NKCC cotransporter and inward rectifying K channels [24], [30] in modulating chloride (and water) transfer across the RPE need to be better understood before pharmaceutical management of myopia can be achieved.

As expected, intravitreal injection of barium severely inhibited refractive compensation to both plus and minus lenses. This could be interpreted as a Ba^2+^ induced suppression of visual function, although electrophysiological evidence suggests otherwise, at least in terms of outer retinal function. In rabbit, acute injection of Ba^2+^ induced an augmentation of the b-wave of the electroretinogram, at least in the short term, while over longer post injection periods the amplitude diminishes [34]. Barium would also be expected to affect aqueous humor secretion and intraocular pressure [35], though our results show no systematic change in anterior chamber depth between the experimental and fellow eyes of the various lens groups. Vitreous chamber depth in all three lens groups was also similar after barium injections, suggesting that nonspecific blockade of potassium channels in the eye inhibits differential ocular growth and refractive compensation to applied defocus. On the other hand our biometric measurements also indicate that barium does not actually stop the eyeball growing.

By comparison the results following raising in the presence of bumetanide are more interesting and less simple to interpret. As expected, we found main effects of lens defocus and drug treatment on refraction and vitreous chamber with a significant defocus/treatment interaction for ocular refraction. However, the general VC elongation seen with bumetanide (except for the higher dose with −10D lens rearing) was slightly unexpected. It appears that a model to explain the data should include a defocus independent elongation effect as well as a defocus dependent effect on refractive compensation and vitreous chamber depth that applies under conditions of negative lens defocus.

We suggest that the most parsimonious interpretation of the success of bumetanide in inhibiting the degree of induced myopia under –ve lens defocus conditions is that the apical RPE and ON-bipolar NKCC1 cotransporters have changed their mean operating conditions, and hence net fluid transport. Currently, it is accepted that when [K+]_SRS_ is raised from 2mM to 5mM, RPE basal to apical ^36^Cl^−^ flux increases dramatically, while the apical to basal ^36^Cl^−^ flow is relatively unchanged, leading to a net secretion rather than absorption of chloride and the slowing of outflow, attributed to the NKCC1 cotransporter and basal NaHCO_3_ [30]. Fluid transport is reduced by 25% under these conditions. The reverse chloride movement was hypothesized to involve a compensatory bicarbonate transport. Fluid flow in the dark [22] and under FD conditions [6] has also been hypothesized to decrease due to the reaccumulation of subretinal potassium. The NaK-ATPase which maintains the potential difference across the RPE must also maintain its activity to maintain the outflow [9]. This activity is dependent on [K]_SRS_, but so also is the cotransport of Na-K-Cl [18]. A bumetanide induced reduction in RPE outflow is also a likely cause of the increase in VC depth observed in the bumetanide injected eyes reared with 0D and+10D lenses, given that aqueous humour production and intraocular pressure changes under bumetanide are minimal (at least in mouse and monkey [36], [37]).

The situation with –ve lens rearing is somewhat different. Defocus together with patterned stimulation results in lowered amplitude of temporal modulation, particularly at high spatial frequencies. Thus, with patterned stimulation, an apical NKCC1 symporter should shunt ions and fluid back and forth between the SRS and the interior of the RPE cell under a sequence of light/dark transitions. The net fluid flow actually generated across the apical membrane of the RPE would be dependant on the modulation of [K] in the SRS and on the time constant for water transport of the receptor mechanism and the relation between water and ion transfer [24]. It has been suggested [6] that defocused pattern stimulation results in a sign-dependant asymmetry in the profile of temporal modulation of the photoreceptors. Hence, as the SRS volume changes are relatively slower than the ionic changes [38], the slower phase of the asymmetric temporal profile of onset or offset will likely drive more fluid than the faster phase. Averaged across the RPE under patterned defocused stimulation, a net reduction in outflow could result if the reverse flow probability was thus biased. The net flow reduction would explain the abnormal axial elongation and myopia. The effect of bumetanide block of the NKCC1 is to prevent this reversal occurring and hence inhibiting the myopic response to –ve lens defocus and also increasing the rate of flow across the RPE to the choroid. In support of involvement of the NKCC1 transporter in these fluid flow changes, it has been reported [38] that bumetanide inhibits the light evoked volume increase in the subretinal space.

Bumetanide, as a selective NKCC1 inhibitor, would be expected to affect the ciliary secretion of Na, K, and Cl ions [36], as well as the targeted apical membrane of the RPE and ON bipolar dendrites. However, as we saw insignificant effects of bumetanide on anterior eye parameters, such effects on the ciliary body can be considered independent of the applied lens defocus. Certainly, light-induced changes in potassium concentration in the subretinal space are enhanced by the application of bumetanide [38], although this may be due in part to the concomitant inhibition of the light-induced SRS volume increase [11]. At high concentrations (0.5mM), bumetanide is reported to inhibit the b-wave of the electroretinogram (ERG) in fish eye cup [39], but the ERG appears little affected at concentrations of bumetanide as used here. Thus, it is unlikely that the effects of blocking the NKCC1 on negative lens defocus, i.e. of inhibiting the development of myopia, are due to any major compromise of outer retinal function. The strong similarity in refractive compensation under +ve lens defocus conditions between bumetanide and saline injected eyes suggests that the bumetanide treated retina is functioning relatively normally under those defocus conditions. As referenced in the introduction, other studies (TTX elimination of spiking neuronal responses, optic nerve section, etc) suggest that considerable damage to inner retinal function can be sustained without compromising refractive compensation ability.

To date there is very little consideration in the literature of the action of bumetanide on the NKCC1 cotransporters on the ON-bipolar dendrites. It would be expected that with bumetanide block, any response to the onset of light, whether in the presence of optical blur or not, would result in a lesser influx of K, Na and Cl ions into the ON bipolars and hence a greater abundance of ions in the extracellular space, increased osmotic gradients and so draw further water out of the cells and increase the need for greater transport of fluid out of the inner retina and across the RPE to the choroid.

Furthermore, it is curious that the effects of bumetanide in primarily inhibiting compensation to –ve lens defocus is reminiscent of similar findings relating alteration of the ON and OFF response and sign-dependant interference with compensation. This interference occurs if the retinal response is environmentally altered through sawtooth illumination [40] or altered through pharmacological blockade of the retinal ON and OFF responses via the use of L- or D-α-aminoadipic acid (LAA and DAA) [41]. Similarly, mouse mutants with an ON pathway defect [42] show increased susceptibility to FDM. The coincidence of findings is more salient because it is the blockade of the ON-response (with LAA) that interferes selectively with ocular growth and refractive compensation to –ve lens defocus.

Thus barium, a potassium channel blocker inhibits compensation to defocus of both signs, while bumetanide, a common loop diuretic approved for human use, demonstrates a selective inhibition on negative lens defocus induced refractive change in the chick. The action of bumetanide appears to combine a defocus-sensitve inhibition of refractive compensation under conditions that would normally lead to myopia, plus a small defocus-insensitive effect on vitreous chamber depth. Given the common channels and symporters on the RPE demonstrated by many animals, including human, there is hope that a rapid development path to a pharmaceutical control of myopia can be established, especially important for the increased incidence observed recently in Asia [43].

## Materials and Methods

### Animals

A total of 139 male hatchling chicks of the Leghorn/Australorp strain were raised in a light and temperature controlled box with *ad libitum* food and water and illumination provided by a static 40W pearl incandescent light bulb on a 12 hour day/night cycle. On day 6, all chicks were anaesthetized with an intramuscular injection of a mixture of ketamine/xylazine (45 mg.kg^−1^/4.5 mg.kg^−1^ i.m.) prior to monocular intravitreal (5 µl) injections of either saline (n = 32), barium chloride (vitreal concentration 5mM in saline, n = 33) or bumetanide (vitreal concentration 10^−5^M (Bum1), n = 31, or 2×10^−6^M (Bum2), n = 43, dissolved in saline and monocular attachment of lenses. All fellow eyes were injected with 5 µl of saline. The initial concentrations of barium and bumetanide used were determined on the basis of previous work [38] and electroretinographic recordings. Doses of drugs are presented as estimated vitreal concentrations. These estimates were calculated using a vitreous volume of 0.5 ml and assuming complete mixing. However, it is acknowledged that mixing will not be rapid, as the diffusion coefficient through the gel vitreous will be considerably less than that through the sol vitreous. Hence the actual release of drug is likely to be more gradual. While the concentration half-life of the two drugs used here has not been quoted, various therapeutic agents demonstrate vitreous half-lives of 2.5–5 day (e.g. [44]).

Chicks were randomly assigned to one of the goggle wearing groups (+/−10D or 0D PMMA human contact lenses). Lenses were attached to a ring of Velcro glued to the periocular feathers. The field of view through the goggle was ∼90^0^. Animals and lenses were checked and cleaned twice daily. To control for batch variation, each batch included chicks from each of the three lens groups to allow more valid comparison.

### Biometry

On Day 10, biometric measurements including retinoscopy and ultrasonography were made on all animals under general anaesthesia (which induces adequate pupillary dilation and negates the need for topical mydriasis). Refractions were recorded as equivalent spherical power. A-scan ultrasonography (Ophthascan: 7MHz) was performed and axial length and vitreous chamber depths were determined. Animals were killed by anaesthetic overdose after biometric measurements were completed.

All animals were raised in accordance with the ARVO Convention and the Australian NHMRC guidelines on Animal Use in Research. Institutional Ethics approval for the experimentation was obtained from the Animal Ethics Committee at La Trobe University, Melbourne, Victoria Australia.

### Data Analysis

Between group statistical comparisons of refractions and axial dimensions were made using Analyses of Variance (ANOVA) and relevant post hoc tests as required. The effects of drugs on the non-lensed fellow eyes were also assessed
